# Transcranial direct current stimulation impairs updating of avoidance-based associative learning

**DOI:** 10.3389/fnhum.2023.1104614

**Published:** 2023-04-24

**Authors:** Mascha van ’t Wout-Frank, Sarah L. Garnaat, Christiana R. Faucher, Amanda R. Arulpragasam, Julia E. Cole, Noah S. Philip, Rebecca D. Burwell

**Affiliations:** ^1^Department of Psychiatry and Human Behavior, Warren Alpert Medical School, Brown University, Providence, RI, United States; ^2^COBRE Center for Neuromodulation, Butler Hospital, Providence, RI, United States; ^3^Center for Neurorestoration and Neurotechnology, VA Providence Healthcare System, Providence, RI, United States; ^4^Department of Cognitive, Linguistic and Psychological Sciences, Brown University, Providence, RI, United States

**Keywords:** tDCS, learning, memory, avoidance, anxiety, brain stimulation, reversal, associative

## Abstract

**Introduction:**

Exposure-based psychotherapies for the treatment of anxiety- and fear-based disorders rely on “corrective” associative learning. Namely the repeated confrontation with feared stimuli in the absence of negative outcomes allows the formation of new, corrected associations of safety, indicating that such stimuli no longer need to be avoided. Unfortunately, exposure-facilitated corrective learning tends to be bound by context and often poorly generalizes. One brain structure, the prefrontal cortex, is implicated in context-guided behavior and may be a relevant target for improving generalization of safety learning. Here, we tested whether inhibition of the left prefrontal cortex causally impaired updating of context-bound associations specifically or, alternatively, impaired updating of learned associations irrespective of contextual changes. Additionally, we tested whether prefrontal inhibition during corrective learning influenced subsequent generalization of associations to a novel context.

**Methods:**

In two separate experiments, participants received either 10 min of 2 mA cathodal transcranial direct current stimulation (tDCS) over EEG coordinate F3 (Experiment 1 *n* = 9, Experiment 2 *n* = 22) or sham stimulation (Experiment 1 *n* = 10, Experiment 2 *n* = 22) while previously learned associations were reversed in the same or a different context from initial learning. Next, to assess generalization of learning, participants were asked to indicate which of the previously seen images they preferred in a novel, never seen before context.

**Results:**

Results indicate that tDCS significantly impaired reversal irrespective of context in Experiment 2 only. When taking learning rate across trials into account, both experiments suggest that participants who received sham had the greatest learning rate when reversal occurred in a different context, as expected, whereas participants who received active tDCS in this condition had the lowest learning rate. However, active tDCS was associated with preferring the originally disadvantageous, but then neural stimulus after stimulus after reversal occurred in a different context in Experiment 1 only.

**Discussion:**

These results support a causal role for the left prefrontal cortex in the updating of avoidance-based associations and encourage further inquiry investigating the use of non-invasive brain stimulation on flexible updating of learned associations.

## 1. Introduction

The ability to flexibly update and adapt our behavior plays a role in anxiety and fear-based disorders. For example, individuals suffering from anxiety and fear-based disorders repeatedly (and often erroneously) respond to feared stimuli as “dangerous.” This anxiety is frequently associated with avoidance of the anxiety-inducing situation and/or performance of safety behaviors (e.g., hand-washing compulsions in case of obsessive-compulsive disorder) to prevent or reduce anxiety. However, such avoidance and safety behaviors are ultimately counterproductive because they sustain the anxiety and prevent the individual from responding flexibly and learning that the feared consequences do not occur (Foa and Kozak, 1986; Salkovskis et al., 1996). Exposure-based psychotherapies aim to tackle this behavioral inflexibility by systematically guiding patients to approach anxiety-provoking situations they would rather avoid. In doing so, exposure therapy provides a corrective learning experience as patients learn that these situations are not as dangerous or threatening as originally thought (Mowrer, 1960; Foa and Kozak, 1986; Beck et al., 2005). This parallels the extinction of fear, and results in the acquisition of new, more adaptive associations and behavioral responses that inhibit excessive or irrational fear (Foa and Kozak, 1986; Harris et al., 2000; Bouton, 2004; Bouton et al., 2006; McConnell and Miller, 2014).

Despite strong experimental support, exposure-based psychotherapy often results in less-than-optimal clinical outcomes; drop-out is high, and many patients experience residual symptoms and a return of symptoms in the months or years following treatment (Heimberg et al., 1998; Craske, 1999; Otto et al., 2000; Dudai, 2006; van Minnen and Foa, 2006; Kindt et al., 2009; Taylor et al., 2012; Rothbaum et al., 2014). Whereas the persistence of symptoms suggests difficulties of the generalization of newly learned associations beyond the treatment setting, the return of fear indicates a failure of extinction-based inhibition over time. One reason why generalization may be limited and inhibition may fail is that unlike initial fear acquisition, which generalizes easily across contexts, subsequent extinction is contextually bound and does not readily generalize to other contexts (Bouton, 1993, 2002, 2004; Bouton et al., 2011; Todd et al., 2012). In other words, a stimulus-response relationship that has been extinguished is considered the “exception to the rule.” This is highlighted by observations in rodents that extinction occurs faster when the extinction context during extinction differs from the context in which initial fear learning took place. Here the change in context signals that something has changed. Yet, a return to the initial (fear) context results in a return of fear, indicating renewal (Bouton and King, 1983; Vansteenwegen et al., 2005), and if a subsequently encountered context is more akin to the initial acquisition context, renewal is enhanced (Todd et al., 2012).

A similar phenomenon has been observed in reversal learning. Like extinction, reversal learning relies on updating contingencies through associative learning. Namely, in standard reversal learning tasks, one of two stimuli is initially associated with a reward after which learned associations are reversed (Cools et al., 2002; Fellows and Farah, 2003; Remijnse et al., 2005). When this reversal takes place in a context that differs from that in which initial learning occurred, reversal learning happens faster (compared to when initial and reversal learning take place in the same context) (Thomas et al., 1985; McDonald et al., 2001). Facilitating the relevant cognitive mechanisms underlying generalization of learning as well as mechanisms to prevent a return of fear might ultimately improve real-world efficacy of exposure therapy and reduce relapse (Boschen et al., 2009).

The prefrontal cortex, and in particular the ventrolateral, dorsolateral, (ventro)medial prefrontal and orbitofrontal cortex, are implicated in stimulus-outcome associative learning and play a critical role in behavioral updating and responding to surprising events when contingencies change (Fletcher et al., 2001; Cools et al., 2002; Clark et al., 2004; Boettiger and Esposito, 2005; Ghahremani et al., 2009; Mitchell et al., 2009; Greening et al., 2011; Levy-Gigi et al., 2011; Izquierdo et al., 2017). For this reason, neuromodulation of the prefrontal cortex might be a promising target to impact the updating of behavior when contingencies change. For example, transcranial direct current stimulation (tDCS) – a type of non-invasive neuromodulation that alters intrinsic neuronal activity using a relatively weak but constant electrical current (Nitsche et al., 2008) – has been found to facilitate implicit learning using a probabilistic classification learning task (Kincses et al., 2004). Additionally, tDCS aimed to inhibit ventrolateral prefrontal cortex functioning was associated with more errors during reversal learning as expected (Albein-Urios et al., 2019) whereas tDCS aimed to excite the medial prefrontal cortex led to increased choice shifting following punishments during reversal learning (Panitz et al., 2022). However, in these prior experiments, context was not manipulated and, as we highlighted above, context affects flexible updating and renewal of previously learned associations, which is relevant when the goal of such research is to contribute to the development of novel treatments for fear-based disorders. To the best of our knowledge, there are no studies examining how tDCS affects updating of contingency-based associations while context is manipulated. Moreover, it remains unknown whether effects of tDCS on updating contingency-based associations also impacts subsequent generalization of learned associations to a novel context.

Therefore, we tested whether inhibition of the left prefrontal cortex using tDCS impacted updating of avoidance-based associative learning when contingencies reversed in both a different context and in the same context as initial learning. Additionally, we examined whether tDCS during contingency reversal affected subsequent generalization of learned associations to a novel context. Given the role of context and the prefrontal cortex discussed above, we predicted (a) that reversal of associations would be easier (i.e., less errors) if the context differed from initial learning than if it remained the same, and (b) that cathodal tDCS would impair the updating of associative learning during reversal. As to generalization of learning, we predicted that last learned rules (during reversal) would generalize to a novel context because of recency effects when reversal occurs in the same context as initial learning. However, when reversal occurs in a different context from initial learning, we hypothesized participants would learn with 50% change that a stimulus can be bad or not, and thus no clear preferences will generalize to a novel context. We remained agnostic as to whether tDCS will affect this generalization. We used tDCS as a temporary non-focal lesion technique, based on the idea that tDCS modulates Hebbian processes (Kronberg et al., 2020) relevant for associative learning-related plasticity.

## 2. Materials and methods

### 2.1. Participants

#### 2.1.1. Experiment 1

Twenty-two healthy participants were recruited from the VA Providence Healthcare System and nearby general community (Providence, RI, USA). Exclusion criteria included any tDCS contra-indications (e.g., current, or history of, neurological disease or closed-head injury; implanted electronic hardware or metal in the cranial cavity; broken skin or other lesions in the area of the electrodes; presence of holes in the skull made by trauma or surgery; pregnancy), current, or history of, psychiatric diagnosis assessed with the Mini Neuropsychiatric Interview Screen for DSM-IV (Sheehan et al., 1998), and current use of psychotropic medications as well as (ab)use of substances and alcohol. To roughly screen for the possibility of difficulties seeing colors (necessary for the experimental tasks) participants completed the first six Ishihara plates (Ishihara, 1918). Because they were recruited from a healthcare environment, participants also completed the General Anxiety Disorder 7 item scale (GAD-7) (Spitzer et al., 2006), the 16-item self-report Quick Inventory of Depressive Symptomatology (QIDS-SR) (Rush et al., 2003) to screen for anxiety and depressive symptoms, respectively, and the Positive and Negative Affect Schedule (PANAS) (Watson et al., 1988) to assess positive and negative affective states. The Providence VA IRB approved study procedures and materials, and consistent with the Declaration of Helsinki written informed consent was obtained prior to onset of any study procedures.

#### 2.1.2. Experiment 2

We performed Experiment 2 to replicate our findings from Experiment 1 in a larger sample. Forty-six participants were recruited from the Brown University campus with the same exclusion criteria as described for Experiment 1. Participants also completed the first six Ishihara plates (Ishihara, 1918), although the PANAS, GAD-7 and QIDS-SR were not administered in this healthy control sample. The Brown University Institutional Review Board separately approved study procedures and materials, and consistent with the Declaration of Helsinki written informed consent was obtained prior to onset of any study procedures.

### 2.2. Avoidance-based association learning

**FIGURE 1:**
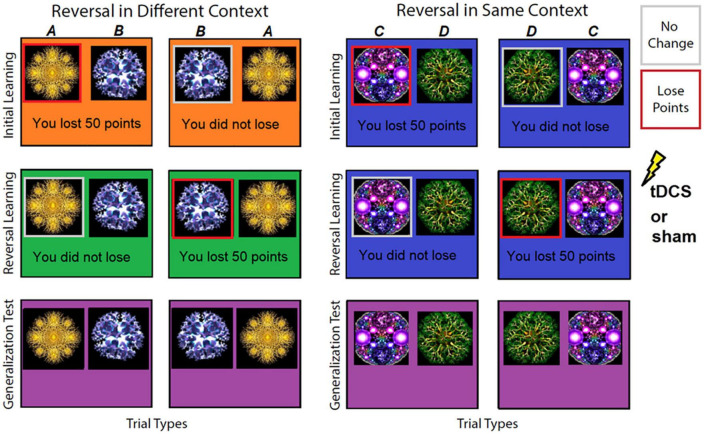
Examples of stimuli and contexts used in the avoidance-based association learning task phases. In the initial learning phase, participants saw two sets of images in either Context 1 (orange background) or Context 2 (blue background). Selecting one image resulted in a 50-point loss on 80% of trials [images **(A,C)**, highlighted in this figure with a red square; square was not shown to participants] or resulted in no points lost [images **(B,D)**, highlighted in this figure with a gray square; square was not shown to participants]. During reversal, these contingencies reversed so that the image that previously resulted in a 50-point loss 80% of the time now resulted in no loss, and vice versa, the image associated with no points lost now led to a 50-point loss on 80% of trials. This reversal occurred in either a different context (Context 3, green background) or the same context (Context 2, blue background) as the initial learning phase. During the generalization test, previously presented image pairs were presented in a never-before- seen Context 4 (purple background) and participants were asked which image they preferred.

All participants, in both experiments, completed an association learning task with the goal to avoid losing points as much as possible (see Figure 1). Because there was never an option to win anything, only to avoid losing, we refer to this task as avoidance based. This avoidance-based approach was selected because it was viewed as more analogous to fear- and anxiety-based disorders, which are generally conceptualized as being maintained by negative (vs. positive) reinforcement, i.e., avoidance. The task consisted of three phases. During the first, *initial learning phase* participants saw two sets of fractal images (A/B) presented in Context 1 (see Figure 1 for images and context color used) or Context 2 (images C/D, see Figure 1 for images and context color used). Selecting images A and C resulted in a 50 point loss and selecting images B and D resulted in no points lost in 80% of trials (*n* = 64). In the remaining 20% of trials (*n* = 16) this was reversed (i.e., selecting images A and C resulted in no points lost and selecting images B and D resulted in 50 points lost) and were classified as incongruent trials. To ensure participants adequately learned the associations, the list of 80 trials total was repeated until participants met the learning criterion of avoiding selecting images A and C by selecting images B and D on ten consecutive trials irrespective of incongruent trials. Once this learning criterion was met, participants started the second phase.

During the second or *reversal learning phase* contingencies reversed, i.e., now images B and D resulted in 50 points lost and images A and C resulted in no loss of points in 80% of trials (*n* = 64). Again, the remaining 20% of trials (*n* = 16) were incongruent and followed initial learning contingencies. In addition, image pair A/B now appeared in new Context 3 (context-dependent reversal), whereas image pair C/D continued to appear in the originally learned Context 2 (context-independent reversal). After meeting the same criterion as for initial learning, participants completed a third phase.

In the third *generalization preference phase* participants saw both previously presented stimuli pairs (A/B and C/D; 16 times each pair for 32 trials total) in a never-before-seen Context 4. This was done to test generalization of previous learning (initial and reversal) and effects of tDCS on generalization to a novel context. Participants were asked to select the image they preferred the most in this novel Context 4. In addition to novel Context 4, participants also indicated their preferences for image A vs. B in previously seen Contexts 1 and 3 (eight times each) and image C vs. D (eight times) in Context 2 to allow preference examination of previously learned contingencies.

Choice feedback on whether 50 points were lost or not, and a running total was provided after each trial in the first and second learning phases of the task, but not the third, choice preference phase. For all three phases (initial learning, reversal, and generalization), images were presented on the screen until participants made a choice with a maximum of 10 s. In between images, participants saw a fixation cross for 1 s. In the initial learning and reversal phases, participants were presented with a 2 s feedback screen after they had selected an image (A or B; C or D).

### 2.3. Transcranial direct current stimulation

Both experiments involved a single-blind, between-subjects design in which participants were assigned to receive either 10 min of 2 mA cathodal tDCS or sham stimulation starting just prior to the beginning of the second, “reversal” learning phase of the task and continued throughout. Current ramp up and down was 30 s each. This allowed the presentation of four trials of the initial learning phase to continue during ramp-up, before seamlessly transitioning to the second, “reversal” learning phase at start of full stimulation.

tDCS was delivered by a built-in rechargeable battery-driven NeuroConn DC-Stimulator Plus device (NeuroConn Inc, Ilmenau, Germany). We used a 1 (cathode) × 1 (anode) unilateral electrode set-up (Nasseri et al., 2015) with each electrode placed in a 5 × 5 cm (25 cm^2^; current density 0.8 A/m^2^) reusable sponge pocket saturated with 0.9% normal saline. Sponges with electrodes were attached to the participant’s head using a rubber headband.

**FIGURE 2:**
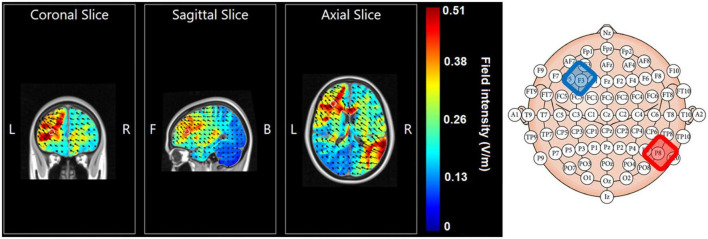
Electrical field modeling of cathode F3 (in blue) – anode P8 (in red) montage using HD Explore, Soterix Medical and their head model.

The cathodal electrode was placed over F3 of the 10–20 EEG electrode coordination system and the anodal electrode was placed over P8. This allowed targeting the left prefrontal cortex with the cathode while preventing inadvertent anodal stimulation over prefrontal regions. Figure 2 displays electrical field modeling results using HD Explore Soterix Medical neurotargeting software and their head model (Kempe et al., 2014) of this electrode montage.

To prevent side effects, the skin under the stimulation sites was lightly cleaned with alcohol and inspected for lesions and abnormalities. Participants were instructed to notify the experimenter of any discomfort and informed that stimulation would be discontinued if discomfort occurred, or if they so wished. To ensure tDCS tolerability to study procedures, all participants initially received 1 mA for 30 s, with a ramp up/down over 30 s each prior to starting the experimental task. Sham stimulation consisted of these same parameters, 1 mA intensity for 30 s with a 30 s ramp up/down each.

### 2.4. Statistical analyses

We used Generalized Linear Models, SPSS version 27, to test whether tDCS Group (two levels), Context (two levels), and their interactions impacted performance accuracy during initial and reversal learning. Because the response variable Accuracy could either be correct or incorrect, we specified a binary logistical model. The variable Participant was entered as a repeated subject variable to adjust for correlations due to repeated observations within participants. Additionally, for both initial learning and reversal phases, we repeated these models by including the variable Trial Number to confirm that performance accuracy would increase throughout the task and to test whether tDCS Group and/or Context affected across trials. We performed these models separately because we recognize that participants completed as many trials as necessary to achieve the predetermined learning criteria and which differed for different participants. Finally, using Generalized Linear Models, we tested whether tDCS Group influenced which stimuli participants would prefer in the final task phase, indicative of whether initial learning or reversal associations generalized, for context-dependent and independent stimulus pairs. Alpha level was set at 0.05.

## 3. Results

### 3.1. Experiment 1

Of the 22 participants, three participants did not complete the learning phases of the task, leaving data from 19 participants for final analyses, with *n* = 9 active tDCS, *n* = 10 sham. See Table 1 for demographics and average accuracy during initial learning and reversal.

**TABLE 1:** Demographics and mean accuracy (standard deviation) across initial and reversal learning trials and contexts for Experiment 1 and 2.

	Experiment 1			Experiment 2		
	**Active *n* = 9**	**Sham *n* = 10**	***p***	**Active *n* = 22**	**Sham *n* = 22**	***p***
Age (years)	44.9 (10.7)	33.1 (15.0)	0.07	26.2 (11.1)	27.8 (10.3)	0.64
Sex (F:M)	4:5	5:5	0.81	13:8*	14:8	0.91
Initial learning	0.79 (0.15)	0.81 (0.10)		0.81 (0.10)	0.83 (0.08)	
Context 1	0.77 (0.17)	0.78 (0.13)		0.79 (0.10)	0.85 (0.09)	
Context 2	0.80 (0.16)	0.83 (0.09)		0.83 (0.11)	0.83 (0.11)	
Reversal learning	0.64 (0.13)	0.72 (0.09)		0.73 (0.13)	0.81 (0.11)	
Context 2	0.64 (0.18)	0.71 (0.10)		0.71 (0.12)	0.78 (0.12)	
Context 3	0.63 (0.10)	0.74 (0.10)		0.75 (0.15)	0.83 (0.12)	

*One participant did not provide an answer for sex assigned at birth. Active vs. sham comparisons indicate *t*-test (age) and Chi-square (sex).

For initial learning, only the main effect for Context was significant (Wald-Chi Square = 7.29, df = 1, *p* = 0.007) and, together with the data reported in Table 1, this indicates that participants made fewer errors in Context 2 compared to Context 1. Given the absence of a significant main effect or interaction with tDCS Group (*p* > 0.05), we conclude that performance did not differ between active and sham groups, as expected during this phase. When repeating the model, now including Trial Number, both the main effect of Context and the interaction Context*Trial Number were significant (Wald Chi-Square = 6.84, df = 1, *p* = 0.009 and Wald Chi-Square = 6.73, df = 1, *p* = 0.009, respectively), demonstrating an increased learning rate in Context 2 compared to Context 1 consistent with the first model. However, the inclusion of Trial Number did not meaningfully improve the goodness of fit of the model based on the Quasi-likelihood under Independence Model Criterion (QIC).

**FIGURE 3:**
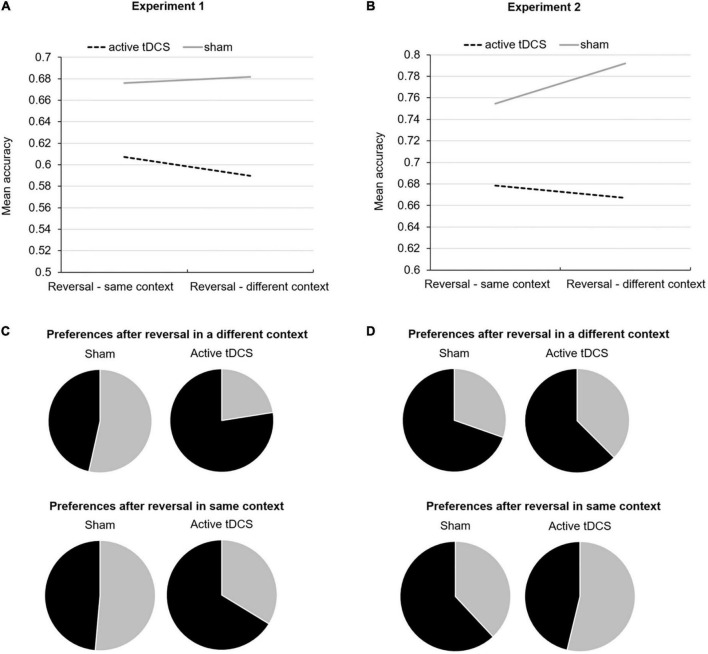
Mean accuracy during reversal learning in the same and different context from initial learning for both active and sham tDCS groups for Experiment 1 **(A)** and Experiment 2 **(B)**. Pie charts reflecting choice preferences in novel Context 4 for stimuli that were initially associated with a loss but reversed to no loss (in black) versus stimuli that were initially associated with no loss but then reversed to loss (in gray) after reversal occurred in a different context and same context from initial learning for Experiment 1 **(C)** and Experiment 2 **(D)**.

For reversal, neither tDCS Group, Context, nor their interaction significantly predicted accuracy (all *p* > 0.05). However, when repeating the model, now including Trial Number, this improved the goodness of fit of the model based on QIC and resulted in a significant main effects tDCS Group (Wald Chi-Square = 8.08, df = 1, *p* = 0.004) and Context (Wald Chi-Square = 5.56, df = 1, *p* = 0.02). Also, the interaction terms tDCS Group*Trial Number, Context*Trial Number, and tDCS Group*Trial Number*Context were significant (respectively, Wald Chi-Square = 5.82, df = 1, *p* = 0.02, Wald Chi-Square = 6.41, df = 1, *p* = 0.01, and Wald Chi-Square = 4.05, df = 1, *p* = 0.04). Data suggest that participants who received active tDCS, compared to sham, had a decreased learning rate which was most pronounced when reversal took place in a different context from initial learning, see Figure 3A.

With respect to generalization preferences in a novel context, tDCS Group significantly predicted preference for the stimulus that no longer resulted in a loss during reversal, when reversal occurred in a different context (Wald Chi-Square = 5.78, df = 1, *p* = 0.02). We did not observe a significant effect of tDCS Group on preferring either stimulus after reversal occurred in the same context (Wald Chi-Square = 1.79, df = 1, *p* = 0.18). One-sample *t*-tests confirmed that, in the novel context, only participants who had received active tDCS preferred the stimulus that no longer resulted in a loss during reversal when reversal had occurred in a different context (*t*(8) = 3.49, *p* = 0.008), which was not true for the sham group who demonstrated no preferences (*t*(9) = −1.53, *p* = 0.16). Participants equally preferred either stimulus in the novel context after reversal occurred in the same context (active tDCS: *t*(8) = −0.38, *p* = 0.72 and sham: *t*(9) = 0.28, *p* = 0.79) (see Figure 3C).

### 3.2. Experiment 2

Out of the 46 participants, two participants did not meet inclusion criteria and did not continue with the experiment, leaving 44 participants for final analysis, with *n* = 22 each allocated to active tDCS or sham. See Table 1 for demographics and average accuracy during initial learning and reversal.

For initial learning, neither tDCS Group nor Context nor their interaction significantly predicted accuracy (all *p* > 0.05), indicating that initial associative learning performance was similar for both tDCS groups and contexts. When adding Trial Number to the model (which did not affect the goodness of model fit based on QIC), the main effect term Trial Number contributed significantly to the model (Wald Chi-Square = 4.82, df = 1, *p* = 0.03), suggesting that performance improved with more trials, as expected.

For reversal, we observed a significant main effect of tDCS Group (Wald Chi-Square = 4.61, df = 1, *p* = 0.03), but no significant main effect of Context or tDCS Group*Context interaction (both *p* > 0.05). Together with data reported in Table 1, this indicates that participants who received active tDCS compared to sham made more errors irrespective of context. When adding Trial Number to the model (which did not affect the goodness of model fit based on QIC), only the three-way interaction tDCS Group*Context*Trial Number was significant (Chi-Square = 4.58, df = 1, *p* = 0.03). Data suggest that while participants who received active tDCS had the lowest reversal learning rate when reversal occurred in a different context, participants who received sham had the highest reversal learning rate when reversal occurred in a different context (see Figure 3B). These observations are consistent with findings reported in Experiment 1.

Regarding generalization preferences, two participants randomized to active tDCS discontinued because of frustrating and did not complete this phase. tDCS Group did not significantly predict stimulus preferences after reversal had occurred in a different context (Wald Chi-Square = 0.56, df = 1, *p* = 0.46) or the same context (Wald Chi-Square = 2.36, df = 1, *p* = 0.13). Irrespective of tDCS Group, one-sample *t*-tests demonstrated that in the novel context, participants preferred the stimulus that no longer resulted in a loss during reversal when reversal had occurred in a different context (*t*(41) = 3.41, *p* = 0.001). Participants across groups equally preferred either stimulus in the novel context after reversal occurred in the same context (*t*(41) = 0.85, *p* = 0.40). See Figure 3D.

## 4. Discussion

This study aimed to test whether cathodal tDCS targeting the left prefrontal cortex, including the ventrolateral and dorsolateral prefrontal cortex, could affect the updating of previously learned avoidance-based associations when this updating occurred in the same or different context from initial learning. This was based on prior literature highlighting the relevance of the ventrolateral and dorsolateral prefrontal cortex for the updating of previous associations (Fletcher et al., 2001; Cools et al., 2002; Clark et al., 2004; Boettiger and Esposito, 2005; Ghahremani et al., 2009; Mitchell et al., 2009; Greening et al., 2011; Levy-Gigi et al., 2011; Izquierdo et al., 2017). Additionally, we tested whether tDCS would impact the subsequent generalization of learned rules to a novel context. This was tested in two separate experiments.

Results from Experiment 2 suggest that cathodal tDCS over EEG coordinate F3 impaired the updating of avoidance-based associative learning irrespective of context. The impeding effect of tDCS on reversal was also observed when the statistical model included the learning rate (i.e., Trial Number) as revealed by a significant three-way interaction with context. Specifically, reversal learning rate was lowest when participants received active tDCS and reversal occurred in a different context, whereas learning rate was highest when participants received sham during this condition (i.e., tDCS + reversal in different context < tDCS + reversal in same context < sham + reversal in same context < sham + reversal in different context). Although the main effect of tDCS was not significant in Experiment 1, perhaps due to low power, when entering learning rate to the model, we observed a similar pattern and three-way interaction as in Experiment 2. Again, indicating that participants who received active tDCS had the lowest learning rate when reversal took place in a different context from initial learning, and the highest learning rate was observed when participants received sham and reversal took place in a different context (with tDCS + reversal in same context and sham + reversal in same context falling in between). This replication of findings in two separate samples, one mostly including individuals from the general population (Experiment 1) and one sample consisting mostly of undergraduate university students (Experiment 2), reinforces the conclusion that tDCS targeting the prefrontal cortex can impair updating of previously learned avoidance-based associations. Our findings are broadly consistent with a prior study in which high definition, cathodal tDCS targeting the ventrolateral prefrontal cortex, but not sham or stimulation of the dorsomedial prefrontal cortex, resulted in higher perseverative errors during probabilistic reversal learning (Albein-Urios et al., 2019).

The interaction between tDCS and context is noteworthy. Characteristically, both in humans and rodents, changes in contextual cues from initial to reversal learning increases the reversal learning rate (Bouton, 1993; McDonald et al., 2001). This is because context functions to retrieve the initially learned stimulus-outcome contingencies hindering updating of the subsequently reversed stimulus-outcome contingencies. Thus, when the context during reversal differs from initial learning, this interference is reduced, facilitating reversal learning. This led to our prediction that reversal would be more difficult when the context did not change, and which might be more prone to interference from tDCS. Although this is not entirely what we observed, i.e., we did not see a significant main effect of context during reversal, the most plausible explanation for the observed three-way tDCS, context, and learning rate interactions is grounded in the observation that the prefrontal cortex, including the dorsolateral and ventromedial prefrontal cortex and their connectivity, are important for context-dependent valuation to guide choices (Rudorf and Hare, 2014). Namely, disruption of the prefrontal cortex due to cathodal tDCS might have specifically impaired the processing of context during reversal updating.

An alternative possible explanation for the effect of tDCS is that tDCS generally disrupted executive functioning, including working memory and/or attention. This is consistent with multiple prior reports of tDCS targeting the left dorsolateral prefrontal cortex yielding altered working memory performance (Fregni et al., 2005; Ohn et al., 2008; Hoy et al., 2013). However, if tDCS impaired executive functioning, we would expect tDCS to mainly impair reversal when it occurred in the same context as initial learning given the greater interference of prior learned associations and thus greater burden on executive functioning, which we did not observe. Likewise, if the negative effect of tDCS was due to distraction (i.e., the physical sensation of tDCS), we would expect to observe an overall effect of tDCS even when learning rate was taken into account.

A second focus of this study was to test whether the manipulation of context and application of tDCS during reversal subsequently influenced generalization of stimuli preferences in a novel (never-before encountered) context. We predicted that last learned associations, i.e., associations learned during reversal, would generalize to a novel context due to recency, and that this would be more pronounced after reversal occurred in the same context as initial learning, compared to when reversal occurred in a different context. We reasoned that the change in context allowed participants to learn, with equal likelihood, that either image would result in a loss (initial learning) in one context and no loss (reversal) in another context. On the other hand, reversal in the same context may “overwrite” learned associations during reversal promoting generalization of last learned rules. Our data appears to partially support this prediction. That is, last learned associations where the stimulus that resulted in a loss during initial learning, but then reversed to no longer result in a loss, was preferred in the novel context. Yet, this was only true after reversal had occurred in a different context. This was true irrespective of tDCS in Experiment 2, but only true for participants who received active tDCS in Experiment 1. These results suggest that, despite tDCS impairing the updating of context-dependent stimulus-outcome associations, the effect of tDCS on the generalization of rules as detected by preferences in a novel context is minimal.

Limitations of the current work include the relatively small sample sizes, especially in Experiment 1. In Experiment 1, our data indicated that participants made significantly fewer errors in Context 2 compared to Context 1 across initial learning trials, and which may reflect differences in perceptual quality between these two contexts. However, this difference between the contexts during initial learning was not replicated in Experiment 2, which included a larger sample. Other limitations are that our task only involved one reversal as opposed to reversing back and forth between primary and secondary learned associations which might have allowed examining the effects of tDCS on renewal, and the administration of a fixed number of trials for each learning phase. Although this was done to allow the duration of the tDCS to match the duration of the task administration, individual variability in reaching the learning criterion might have led to over practice in some individuals and could have influenced subsequent task phases. Finally, we applied a constant electrical current (2 mA) which likely resulted in variable electrical field values in participants (Evans et al., 2020). It is possible that individual electrical dosimetry might yield different outcomes and future studies are needed to address this general issue concerning tDCS.

Nevertheless, our results support a causal role for the left prefrontal cortex in the updating of avoidance-based associations, a neurocognitive process that lies at the heart of exposure-based psychotherapy for anxiety and fear-based disorders. These findings encourage further exploring the effects of non-invasive brain stimulation on flexible updating of learned associations to ultimately augment exposure-based treatments for anxiety and fear-based disorders.

## Data availability statement

Data will be made available upon request following VA regulations.

## Ethics statement

The studies involving human participants were reviewed and approved by the VAPHS IRB (Experiment 1) and Brown University IRB (Experiment 2). The patients/participants provided their written informed consent to participate in this study.

## Author contributions

MW-F designed the experiment, collected and analyzed the data, and wrote the manuscript. SG designed the experiment and wrote the manuscript. CF collected and processed the data and wrote the manuscript. AA and NP interpreted the data and wrote the manuscript. JC processed the data and wrote the manuscript. RB designed the experiment, interpreted the data, and wrote the manuscript. All authors contributed to the article and approved the submitted version.
